# Combating COVID-19 Pandemic in Nepal: Ethical Challenges in an Outbreak

**DOI:** 10.31729/jnma.4959

**Published:** 2020-04-30

**Authors:** Aarati Shah, Ramesh Prasad Aacharya

**Affiliations:** 1 Medical Education Commission, Sanothimi, Bhaktapur, Nepal

**Keywords:** *COVID-19*, *ethics*, *public health*, *strategy*

## Abstract

Pandemic outbreak of COVID-19 is the largest of its kind of this century. All countries throughout the globe are trying their best to contain the disease and eliminate at the earliest. Efforts are continuing to improve the outcome of the infection in terms of minimizing the morbidity and mortality. As a public health strategy every state has the responsibility of protecting the health of the community and such measures includes the preventive measures like social distancing or even lockdown of the state as a whole restricting the movement of the people, diagnostic measures like testing the suspects, contact tracing and isolation of the patients. Treatment of the infected requires decisions in resource constraint situation particularly ICU beds and ventilators. In the meantime, protecting doctors, nurses, other health workers as well as frontline workers need personal protective equipment (PPE) which is a scarce commodity. While doing so there might be a compromise in the individual autonomy, privacy, confidentiality, and social justice for the beneficence for the larger community. This is an attempt to explore the ethical quandaries in relation to combating COVID-19 in Nepal by relating the issues with the principles of biomedical ethics.

## INTRODUCTION

Since the outbreak in Wuhan city of China in December 2019, COVID-19 has spread over the globe as a pandemic significantly affecting day to day human life posing challenges and forcing entire world in a status of lockdown. In the present context, every state has the responsibility to protect the health of its population which needs crucial public health strategies and interventions accordingly.^1^ As of 14th April 2020, 15 sporadic immigrant cases and one local spread have been detected in Nepal indicating the alarm entering from phase 1 to phase 2 needing rigorous enforcement of certain measures to protect the Nepalese population from further transmission of the disease in the community.

The global spread is demanding huge public health and prompt medical responses which include detection of sources, testing of anticipated positive cases, contact tracing, and combating further spread. Nepal government has imposed travel restrictions as well as lockdown of the country sealing land borders with India and China and halting all the national and international flights. In the meantime, spreading health education for social distancing, avoiding handshakes and hugs, and doing “Namaste” is becoming more acknowledged as a way of greeting.

Protection of the health of the population needs crucial public health planning and rigorous enforcement of certain public health measures. As a strategic planning phase 1, one has to get ready for the surveillance system to detect suspected cases of COVID 19 infection and isolate them. Following with contact tracing and putting suspected cases in quarantine for 14 days. The lessons learned from Italy suggest that the right to individual liberty and the right to mobilize, communicate or socialize have to suspend, to prevent the progression of stage 1 to other stages.^2^ China succeeded in combating the situation by effective enforcement of restriction of movement, quarantine, and isolation and labeled it as the ‘China model’.^3^

News reports of people being assaulted when they violate the lockdown by coming out of their homes by law enforcement agencies are starting to surface. Migrant workers trying to enter Nepal from India are denied entry. People are taking desperate measures like crossing rivers to enter into Nepal. Those who are caught have been arrested of benefit to the community, but are deprived of their autonomy. The restrictions made on the individual liberties should meet the principle of proportionality and it should not exceed to the extent it exceeds anticipated risk.^4^

The health workers assigned on duty without PPE in the frontlines are at increased risk not only to themselves but also to their family members and other patients. Thus, it poses an ethical issue of harming others. In addition, there is fear and hesitancy in care providers who are refusing to attend the patients. Patients with fever are being stigmatized for COVID-19 even though they have tested negative. At times, patients suspected of COVID-19 died in isolation wards but so far all of them were negative for the disease. However, obviously the dead body could not be cremated until receiving the test report. Even after that their funeral, their families have been boycotted by near and dear ones.

Distribution of scarce medical supplies including PPE to government institutions only has augmented the undue referral or even refusal of patient care. The question is whether the ethics have been violated in the war of combating the COVID-19 pandemic?

## DISCUSSION

After the declaration of a pandemic by the World Health Organization (WHO), all nations were alerted. Nepal had to be more vigilant due to its border to the originating country China and open border with the giant neighbor India. Even the number of cases is still low, a country like Nepal could not ignore the context.

### Autonomy

Several measures are taken to tackle the COVID-19 pandemic, like banning public gatherings, stay-at-home orders, quarantine of returning travelers, and isolation of patients, jeopardized not only the ethical principle of ‘respect for autonomy’ but also the basic human right. However, individual freedom had to be balanced against compelling public health need.^1^ Generally, ethical values in outbreaks or pandemic involve tradeoffs between the individual versus public health interest.^5^ Stay-at-home popularly called as ‘lockdown’ was enforced in Nepal as per the Contagious Disease Act, 1962.

Isolation of the patient and contact tracing carried the risk of violation of the ethical principles of ‘respect for autonomy’ as every suspected case must be reported with personal identifiers, and as per the ethics of pandemics, consent from the individual is not mandatory. This means it is ethically justifiable to breach the issues of privacy and confidentiality because the particular person has to be identified, reported, and isolated to reduce significant harm to the community keeping in mind this overriding should be kept to the minimal.^6^

Like other parts of the world, punitive measures eroded public trust.^7^ Providing appropriate information, truth-telling, and transparent actions augmented the sense of civic responsibilities, and volunteerism was observed in the public.^8–11^ Risk communication and community engagement is a critical component of the response to COVID-19 that helps people make the right decisions about how to protect themselves when to seek care, and to avoid contributing to panic about the disease^12^. Managing the ‘infodemic’ and maintaining trust in public health authorities is critical to the ongoing management of the outbreak.^13^ Trust begins with communication, and communicating information during outbreaks is challenging, especially as our knowledge of the disease is rapidly evolving.^14^

### Beneficence

Like in many countries, separate clinics named either ‘Fever Clinic’ or ‘COVID-19 Outpatients Clinic’ were established in the hospitals so as to identify and triage COVID-19 cases. A suspected case would be shifted to an isolation ward. These measures not only ease the patients but also protect other patients and healthcare providers. Though there was an acute shortage of PPE, gradually specific clinics and wards received them in fair quantities. In any given context, a mechanism of supporting vulnerable individuals to mitigate the effects of these measures has to be ensured.^15,16^ In addition, containment measures must not be a subterfuge for discrimination.^8^

### Non-maleficence

The government decision to implement lockdown adversely affected the poor, the marginalized, and the vulnerable population (for example, daily wage workers, people with diseases requiring regular treatment and follow-up, etc). Even though the government tried to alleviate these deprivations of basic daily needs through local government, it was not possible to reach all those who need it.

Categorical exclusion for care based on sex, race, religion, intellectual disability, citizenship, social status, etc. is morally irrelevant and must be avoided in any circumstance. Categorical exclusions are not necessary because less restrictive approaches can be explored. For example. allowing all patients to be eligible and giving priority to those most likely to benefit.^9^ Some of the incidents included discrimination against doctors, nurses, and other health care professionals. They were asked by the landlords to leave their rented accommodation. However, in the level of society, the health care professionals were highly appreciated for their services irrespective of their risks of COVID-19. Labeling the special clinic as “Fever Clinic” created discrimination of the patients presenting with fever because all patients with fever were regarded as COVID-19 and there was denial or inappropriate management of febrile patients.

### Justice

Non-critical medical care and treatments were limited or even unavailable in some places. Rationing of health care was necessary because the crisis was beyond the existing capacity. In such a situation the ethical dilemma is on deciding who should get the priority for care? ‘Greatest good for the greatest number’ seems logical but at times cannot address some ethical considerations. For example, the allocation of scarce beds in the intensive care units or ventilators for critically ill patients which are generally prioritized to those most likely to be discharged alive after the treatment.^9^ While caring for a case of COVID-19, mortality among elderly is an issue and thus, there is a risk of age-related discrimination for care. In this case, prioritization should actually not be based on age-wise categorical exclusion but based on chances of survival and the opportunity to live through the stages of life - childhood, young adulthood, middle age, and old age.^17^

Another global problem of ethical concerns in the allocation of resources is the availability and distribution of PPE. During a pandemic, keeping medical workers safe is very important. Of course, there is increased vulnerability but the sense of safety needs to be ensured with standard safety measures. Awareness among the medical workers particularly in terms of levels of protection is required. Inappropriate use of PPE by the leaders and nonmedical staff has also reported in the press and must be condemned.

After the blockade of the Nepal-India border particularly following the lockdown, hundreds of people were stranded at both sides of the border. At least one of them turned out to be positive for COVID-19 and thus, contact tracing in this particular case was impossible. Capacity building and collaboration between countries are needed to strengthen the system for the control of any Public Health Emergency of International Concern (PHEIC).^18–21^ International health regulations of the World Health Organization (WHO) might need to be upgraded to global regulations for an international response.

### WAY FORWARD

Strategies like social distancing by the lockdown, quarantine or isolation are some of the public health measures which are enforced to the public for the benefit of the community in a situation of pandemics. It might impose ethical issues of compromise in the autonomy of the individual, ban on the right of transportation, loss of job or income, inaccessibility of marginalized population to basic needs like food, shelter or treatment. Similarly, judicious allocation of limited resources like PPE, test kits, beds, medical equipment, ventilators will help in mitigating the situational crisis and lessening the chances of posing social injustice to the vulnerable population. Beneficence to the large community is acceptable although there might be ethical challenges compromising individual dignity and rights.

## Conflict of Interest

**None.**

## References

[1] Gostin LO, Wiley LF (2020). Public Health ethics and law [Internet].

[2] Remuzzi A, Remuzzi G (2020). COVID-19 and Italy: what next?. Lancet.

[3] Zhu H, Wei L, Niu P (2020). The novel coronavirus outbreak in Wuhan, China. Glob Health Res Policy.

[4] Ethics Resources on The Coronavirus (COVID-19) The Hastings Centre.

[5] Guide to the ethics of surveillance and quarantine for novel coronavirus (2020). [Internet].

[6] Fisher D, Wilder-Smith A. (2020). The global community needs to swiftly ramp up the response to contain COVID-19. Lancet.

[7] Gostin LO, Wiley LF (2020). Governmental Public Health Po wers During the COVID-19 Pandemic: Stay-at-home Orders, Business Closures, and Travel Restrictions. JAMA.

[8] Gostin LO, Friedman EA, Wetter SA (2020). Responding to Covid-19: How to Navigate a Public Health Emergency Legally and Ethically. Hastings Cent Rep.

[9] White DB, Lo B (2020). A Framework for Rationing Ventilators and Critical Care Beds During the COVID-19 Pandemic. JAMA.

[10] Rosenbaum L (2020). Facing Covid-19 in Italy - Ethics, Logistics, and Therapeutics on the Epidemic's Front Line. N Engl J Med.

[11] (2020). Rapid outbreak response requires trust. Nat Microbiol.

[12] Risk communication and community engagement readiness and response to coronavirus disease (COVID-19): Interim Guidance (2020). [Internet].

[13] Zarocostas J (2020). How to fight an infodemic. Lancet.

[14] Childress JF, Faden RR, Gaare RD, Gostin LO, Kahn J, Bonnie RJ (2002). Public health ethics: mapping the terrain. J Law Med Ethics.

[15] Poole DN, Escudero DJ, Gostin LO, Leblang D, Talbot EA (2020). Responding to the COVID-19 pandemic in complex huma nitarian crises. Int J Equity Health.

[16] Berger ZD, Evans NG, Phelan AL, Silverman RD (2020). Covid-19: control measures must be equitable and inclusive. BMJ.

[17] Williams A (1997). Inter generational equity: an exploration of the ‘fair innings’ argument. Health Econ.

[18] Nakazawa E, Ino H, Akabayashi A (2020). Chronology of COVID-19 cases on the Diamond Princess cruise ship and ethical considerations: a report from Japan. Disaster Med Public Health Prep.

[19] Kandel N, Chungong S, Omaar A, Xing J (2020). Health security capacities in the context of COVID-19 outbreak: an analysis of International Health Regulations annual report data from 182 countries. Lancet.

[20] Jee Y (2020). WHO International Health Regulations Emergency Committee for the COVID-19 outbreak. Epidemiol Health.

[21] Gostin LO, Katz R (2016). The International Health Regulations: The Governing Framework for Global Health Security. Milbank Q.

